# Digital chest drainage system versus traditional chest drainage system after pulmonary resection: a systematic review and meta-analysis

**DOI:** 10.1186/s13019-019-0842-x

**Published:** 2019-01-18

**Authors:** Hong Wang, Wenbin Hu, Liang Ma, Yiran Zhang

**Affiliations:** 1 Department of Surgery, Zhejiang University Hospital, Zhejiang University, Hangzhou, China; 2 Department of Cardiothoracic Surgery, First Affiliated Hospital, Zhejiang University School of Medicine, Hangzhou, 310003 China

**Keywords:** Digital chest drainage system, Pulmonary resection, Postoperative care

## Abstract

**Background:**

Several randomized controlled trials (RCTs) and observational studies have compared the efficacy of digital chest drainage system versus traditional chest drainage system. However, the results were inconsistent.

**Methods:**

We searched the Web of Science and Pubmed for observational studies and RCTs that compared the effect of digital chest drainage system with traditional chest drainage system after pulmonary resection. Eight studies (5 randomized control trails and 3 observational studies) comprising 1487 patients met the eligibility criteria.

**Results:**

Compared with the traditional chest drainage system, digital chest drainage system reduced the risk of prolonged air leak (PAL) (RR = 0.54, 95%CI 0.40–0.73, *p* < 0.0001), and shortened the duration of chest drainage (SMD = − 0.35, 95%CI -0.60 - -0.09, *p* = 0.008) and length of hospital stay (SMD = − 0.35, 95%CI -0.61 - -0.09, *p* = 0.007) in patients after pulmonary resection.

**Conclusions:**

Digital chest drainage system is expected to benefit patients to attain faster recovery and higher life quality as well as to reduce the risk of postoperative complications. Further RCTs with larger sample size are still needed to more clearly elucidate the advantages of digital chest drainage system.

## Introduction

Alveolar air leak is one of the most frequent complications after pulmonary resection, which happened in up to 50% patients [1]. Prolonged air leak (PAL), defined as an air leak persisting more than 5 days by the Society of Thoracic Surgeons Database, occurred in approximately 8–15% patients after pulmonary resection [2]. It had been shown that PAL was associated with longer length of hospital stay, increased hospitalization costs, increased risk of empyema, and other possible cardiopulmonary complications [3, 4]. Thus, better approaches for postoperative care are needed to optimize the recovery of patients after pulmonary resection.

Traditionally, an analog chest drainage system was used for assessment of the postoperative air leak. However, the traditional chest drainage system has several limitations. On the one hand, it measures air leak in a subjective manner by observing bubbling in the water chamber, thus interobserver disagreement is frequent, and small air leaks are difficult to determine. On the other hand, the suction pressure of the traditional chest drainage system may deviate from the set level due to the position of the water chamber. Recently, a digital chest drainage system has been developed to solve these problems, which uses digital sensors to monitor air flow and pleural pressure continuously [5]. With the digital chest drainage system, the pleural pressure can be constantly maintained by physicians independent of the device position, and postoperative air leak can be evaluated objectively. Several randomized controlled trials (RCTs) and observational studies have compared the efficacy of digital chest drainage system versus traditional chest drainage system. However, no meta-analysis has been conducted to pool the results of these clinical trials so far.

A meta-analysis and systematic review was performed to compare the efficacy of digital chest drainage system with traditional chest drainage system.

## Materials and methods

### Eligibility criteria

The inclusion criteria of literatures in the present meta-analysis were (i) observational studies or randomized trials, (ii) adults (≥18 years) undergoing pulmonary resection (including lobectomy, segmentectomy, and wedge resection), (iii) studies comparing digital chest drainage system with traditional chest drainage system, (iv) end points included prolonged air leak (defined as air leak duration ≥5 days), duration of chest drainage and length of hospital stay. The exclusion criteria were: (i) case reports or review articles, (ii) articles written in non-English language.

### Search strategy

A literature search was conducted using the Web of Science and Pubmed to identify relevant literatures published through January 2018. The search term used was “digital thoracic drainage”. Two authors (H Wang, W Hu) independently applied the eligibility criteria to screen the literature search results, and the reference lists of the included literatures were screened again for more potential studies. Any divergence was resolved by a third reviewer (Y Zhang).

### Data abstraction and quality assessment

Data abstraction and quality assessment were conducted using methods described previously [6]. Briefly, two authors (H Wang, W Hu) independently extracted the data and evaluated the quality of the included studies. Any divergence was resolved by a third reviewer (Y Zhang). The following data was obtained from each study: author, nationality, publication time, number of patients in each study group, study design, baseline characteristics and clinical end points. Two authors evaluated the quality of the included literatures independently. The Newcastle-Ottawa scale was applied to assess the quality of observational studies (http://www.ohri.ca/programs/clinical_epidemiology/oxford.asp). The following 3 aspects of an observational study were evaluated using Newcastle-Ottawa scale: (1) the selection of the study cohort (or cases/controls), (2) the comparability of the cohorts (or cases/controls) and (3) the outcome assessment for a cohort study, or the determination of the exposure for a case-control study. The Jadad scale was used to assess the quality of randomized trials [7]. The following aspects of a randomized study were evaluated by the Jadad scale: randomization, double blinding, withdrawals and dropouts. A study was considered as high-quality if its score ≥ 3. The present meta-analysis and systematic review was conducted following the PRISMA guidelines [8].

### Statistical analysis

The statistical analysis was performed according to previously described methods [6]. Briefly, we use Review Manager 5.2 (RevMan 5.2®, Nordic Cochrane Center and Copenhagen, Denmark) to conduct the meta-analysis. We calculated Risk ratios (RRs) with a 95% confidence interval (CI) using the Mantel–Haenszel method in order to compare the risk of prolonged air leak between digital chest drainage system and traditional chest drainage system. To investigate continuous measures (duration of chest drainage and length of hospital stay), we calculated standardized mean difference (SMD) with a 95%CI using the Inverse Variance method. Forest graphs were applied to present the meta-analysis results. The statistical heterogeneity of included literatures was assessed by I^2^ statistic. I^2^ values ≤50%, 50–74 and ≥ 75% indicate low, moderate and high heterogeneity [9]. A fixed-effects model was chosen to perform the meta-analysis when the I^2^ value was ≤50%. A random-effects model was chosen when the I^2^ value was > 50%. *P*-value < 0.05 was considered as statistically significant.

## Results

### Description of the included studies

**Fig. 1:**
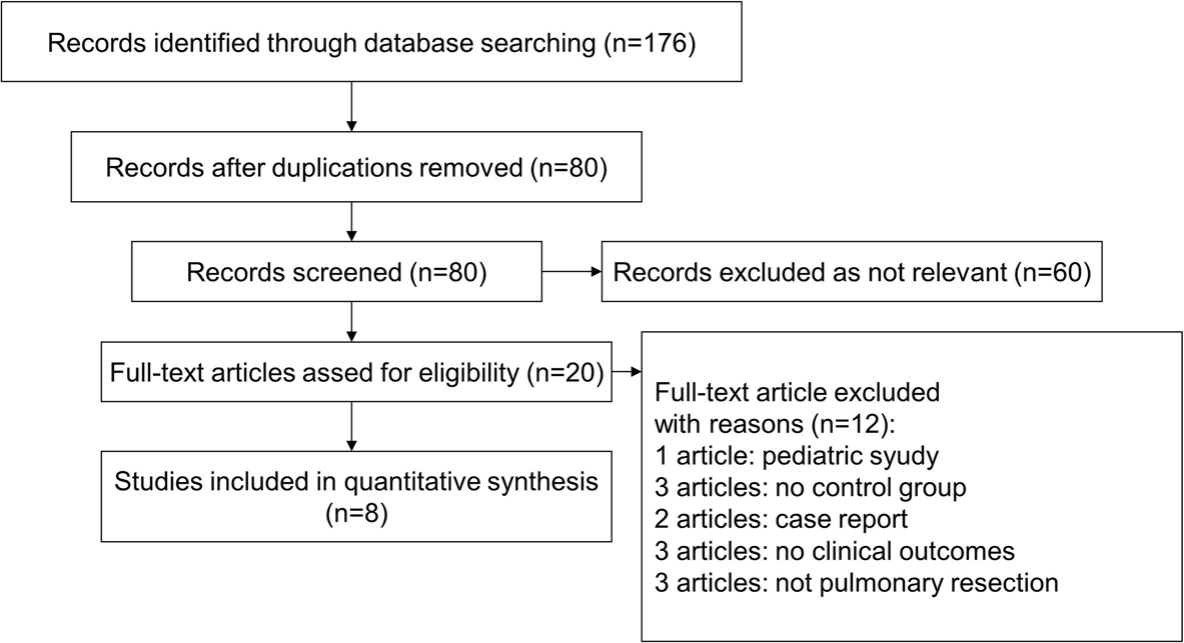
Flow diagram of the article selection process in this meta-analysis

There were 80 articles identified through the literature search process. Among the 80 articles, 60 articles were excluded as not being relevant. The remaining 20 studies were assessed for eligibility, and 8 studies (5 randomized control trails and 3 observational studies) [10–17] comprising 1487 patients met our eligibility criteria and were included in this meta-analysis (Fig. 1). Among the 1487 patients, digital chest drainage systems were used in 720 patients, and traditional chest drainage systems were used in 767 patients. The characteristics of the studies included are shown in Table 1. The baseline characteristics of the patients are shown in Table 2.

**Table 1:** Characteristics of Included Studies

Studies	Year	Country	Number of patients		Study design	Study quality
			Digital	Traditional		
Takamochi	2017	Japan	135	164	RCT	High (5)
Waele	2017	Canada	53	50	RCT	High (5)
Miller	2016	USA	20	40	POS	S3; C1; O3
Shoji	2016	Japan	112	121	ROS	S3; C1; O3
Filosso	2015	Italy	40	40	POS	S3; C1; O3
Gilbert	2015	Canada	87	85	RCT	High (5)
Pompili	2014	International	191	190	RCT	High (5)
Brunelli	2009	Italy	82	77	RCT	High (4)

*RCT* randomized control trial, *ROS* retrospective observational study, *POS* prospective observational study

**Table 2:** The baseline characteristics of the patients

Studies	Male (%)		Age (years)		VATS (%)		Lobectomy (%)	
	Digital	Traditional	Digital	Traditional	Digital	Traditional	Digital	Traditional
Takamochi 2017	51.8	48.7	66.6 ± 12.6	67.9 ± 10.9	NA	NA	77	81.1
Waele 2017	48.1	51.9	68.5 ± 10.3	64.8 ± 10.6	56.2	43.8	50.8	49.2
Miller 2016	55	60	63 (48–77)	63 (52–79)	100	100	85	85
Shoji 2016	69	86	67 (20–88)	65 (19–87)	NA	NA	66	62
Filosso 2015	60	60	69 ± 7.9	67 ± 8.3	NA	NA	80	78
Gilbert 2015	36.5	36.8	68 (60–72)	68 (60–75)	73.6	70.6	70.1	83.5
Pompili 2014	49	55	66.5 ± 12.1	65.9 ± 10.2	82	80	83	88
Brunelli 2009	70	77	66.1 ± 12.8	67.3 ± 8.4	NA	NA	NA	NA

*VATS* video-assisted thoracic surgery, *NA* not available

### Prolonged air leak

**Fig. 2:**
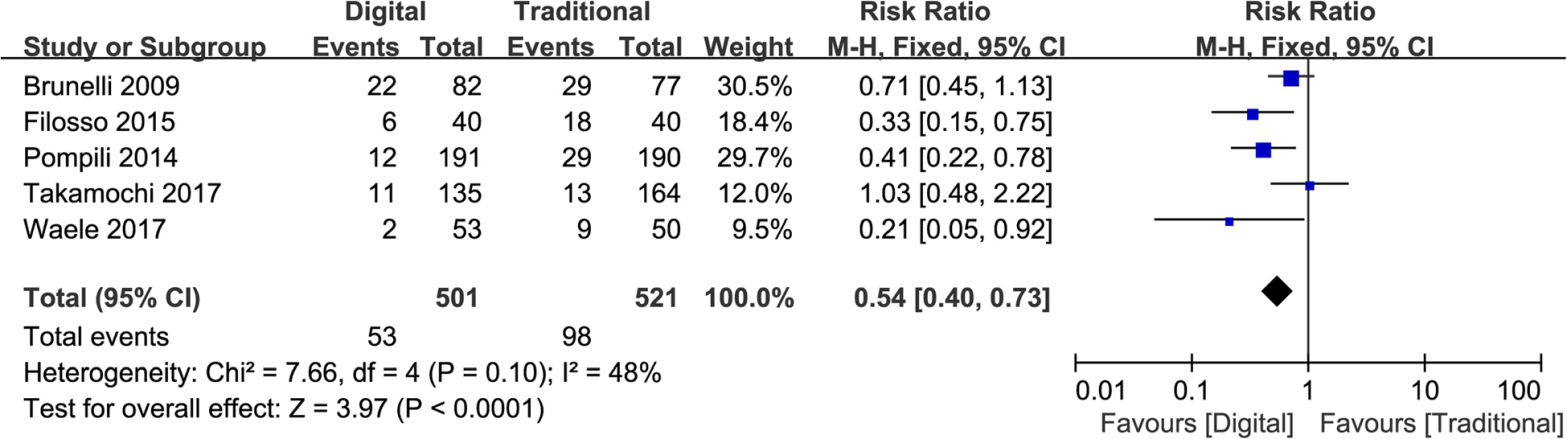
Forest graph presenting prolonged air leak. 95%CI: 95% confidence interval

Five studies evaluated the incidence of prolonged air leak [11, 12, 14, 16, 17]. Digital chest drainage system significantly reduced the risk of prolonged air leak compared with traditional chest drainage system (RR = 0.54, 95%CI 0.40–0.73, *p* < 0.0001; Fig. 2). No significant heterogeneity was observed in the pooled group of studies (I^2^ = 48%, Chi^2^ = 7.66, *p* = 0.10; Fig. 2). Among the five studies, four studies were randomized control trials [11, 12, 16, 17], in this subgroup, the risk of prolonged air leak was still lower in digital group than in traditional group (RR = 0.59, 95%CI 0.43–0.82, *p* = 0.002). One study was observational study [14], which the risk of prolonged air leak was also lower in digital group than in traditional group (RR = 0.33, 95%CI 0.15–0.75, *p* = 0.008).

### Duration of chest drainage

**Fig. 3:**
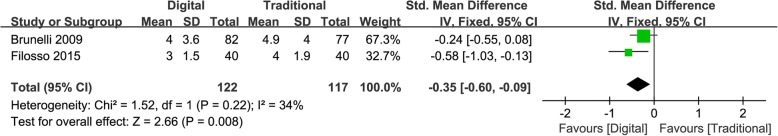
Forest graph presenting duration of chest drainage. 95%CI: 95% confidence interval

Eight studies reported the duration of chest drainage [10–17]. Among the 8 studies, two studies presented the data in a mean ± standard deviation form [11, 14], and meta-analysis of these two studies suggested that digital chest drainage system significantly reduced the duration of chest drainage compared with traditional chest drainage system (SMD = − 0.35, 95%CI -0.60 - -0.09, *p* = 0.008; Fig. 3). The mean or median duration of chest drainage in the 8 studies were summarized in Table 3.

**Table 3:** Duration of chest drainage and hospital stay

Studies	Duration of chest drainage (days), digital vs traditional	Length of hospital stay (days), digital vs traditional
Takamochi 2017 ^b^	2.0 vs 3.0, *p* = 0.149	6.0 vs 7.0, *p* = 0.548
Waele 2017^a^	2.3 vs 2.5, *p* = 0.055	4.8 vs 4.9, *p* = 0.403
Miller 2016 ^b^	3.7 vs 5.3, *p* = 0.01	4.1 vs 5.6, *p* = 0.05
Shoji 2016 ^a^	2.7 vs 3.7, *p* = 0.004	NA
Filosso 2015 ^a^	3 vs 4, *p* = 0.0009	7 vs 8, *p* = 0.0385
Gilbert 2015 ^b^	4.9 vs 5.6, *p* = 0.11	6.0 vs 6.0, *p* = 0.36
Pompili 2014 ^a^	3.7 vs 4.7, *p* = 0.001	4.6 vs 5.6, *p* < 0.0001
Brunelli 2009 ^a^	4.0 vs 4.9, *p* = 0.0007	5.4 vs 6.3, *p* = 0.007

*NA* not available

^a^mean

^b^median

### Length of hospital stay

**Fig. 4:**
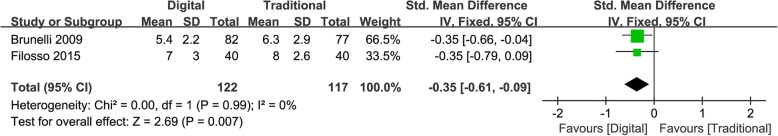
Forest graph presenting length of hospital stay. 95%CI: 95% confidence interval

Seven studies reported the length of hospital stay [11–17]. Among the 7 studies, two studies presented the data in a mean ± standard deviation form [11, 14], and meta-analysis of these two studies suggested that digital chest drainage system significantly reduced the length of hospital stay compared with traditional chest drainage system (SMD = − 0.35, 95%CI -0.61 - -0.09, *p* = 0.007; Fig. 4). The mean or median length of hospital stay in the 7 studies were summarized in Table 3.

## Discussion

In the present meta-analysis and systematic review, we found that compared to traditional chest drainage system, digital chest drainage system reduced the risk of incidence of prolonged air leak, and shortened the duration of chest drainage and length of hospital stay in patients after pulmonary resection.

PAL remains a common complication after pulmonary resection. Several studies had shown that PAL was associated with longer hospital stay and more hospital costs [18, 19]. It was also suggested that PAL was associated with an increased rate of postoperative morbidity, such as empyema, fever and pneumonia [20]. The risk of PAL can be predicted by several preoperative and intraoperative factors such as a low predicted postoperative forced expiratory volume in 1 s (ppoFEV1), pleural adhesions and upper lobectomy [20]. Besides, improvement in postoperative chest drainage system is an important approach to reduce PAL rate and accelerate the recovery.

There are several advantages of digital chest drainage system in management of patients after pulmonary resection. First of all, the digital system can regulate its suction pressure according to the condition in the pleural cavity, and the pleural pressure can be maintained at a preset level within 0.1 cmH_2_O. It had been shown that wide oscillation in early postoperative pleural pressure was associated with a higher incidence of PAL [21]. Thus, the digital chest drainage system may promote the sealing of air leaks by stabilizing the pleural pressure with minimal oscillation. Second, the digital system measures the extent of air leak objectively, and the historical data can be exported and reviewed. Thus, the digital chest drainage system reduces the interobserver variability, and helps medical personnel decide when to remove the chest tube more accurately. It had been proved by several clinical trials that digital chest drainage system not only reduced interobserver variability between different groups of medical staffs (surgeons, residents and nurses) [22], but also between surgeons with comparable experience [23]. The reduced interobserver variability leads to shorter duration of chest drainage and length of hospital stay. Furthermore, the digital system facilitates an early patient mobilization and improves postoperative physiotherapy, which can reduce the risk of secretion and pneumonia, and facilitates pulmonary re-expansion [14]. Finally, the digital device such as the Thopaz chest drain system (Medela Switzerland) can serve as a portable suction unit, and patients can be discharged earlier with this system [24].

Since the traditional chest drainage system is subjective and inaccurate in judging air leak, there is risk of removing the chest tube prematurely. In that situation, chest tube reinsertion is needed. A clamping test had traditionally been taken to prevent this error. It had been suggested by Takamochi et al. that over 50% patients underwent clamping test before removing the chest tube in the traditional group, while none clamping test was taken in the digital group [17]. It had also been shown by Gilbert et al. that chest tube reinsertions for worsening pneumothorax or subcutaneous emphysema after chest tube removal occurred only in the traditional group, none chest tube reinsertion happened in the digital group [13].

Some clinicians hypothesized that the intermittent suction pressure provided by the digital system may reduce the pressure gradient for fluid filtration across the pleural membrane and lighten the inflammatory response. However, the study conducted by Waele et al. did not find such effect [16].

Apart from objective outcomes, subjective outcome (such as patient satisfaction) is also important in evaluating chest drainage system. The multicenter study conducted by Pompili et al. evaluated the patient satisfaction with digital chest drainage system, and the result suggested that patients in the digital group had a more positive perception of the chest drainage, which was associated the comfort, portability and convenience of the digital system [12].

Recently, a meta-analysis of randomized controlled trials conducted by Zhou et al. suggested that digital chest drainage following pulmonary surgery reduced the duration of chest tube placement, length of hospital stay, air leak duration and postoperative cost [25]. Compared to their study, the present systematic review focused on patients after pulmonary resection, and obtained similar results. Another recent study discussed the postoperative air leak pattern in lung cancer patients after pulmonary resection with the help of digital chest drainage system, which suggested that the detailed air leak pattern can be used to predict the duration of air leakage and chest tube drainage [26].

## Conclusion

The present systematic review shows that digital chest drainage system is expected to benefit patients to attain faster recovery and higher life quality as well as to reduce the risk of postoperative complications. Further RCTs with larger sample size are still needed to more clearly elucidate the advantages of digital chest drainage system.

## Abbreviations

CI: Confidence interval
PAL: Prolonged air leak
ppoFEV1: Predicted postoperative forced expiratory volume
RCT: Randomized controlled trial
RR: Risk ratio
SMD: Standardized mean difference
